# Brain Disease

**Published:** 1875-04

**Authors:** 


Brain Disease.—In brain disease, so common at the present day, there is no practice so valuable—as an adjunct to a perfect cure—as reasonable eating. By this we mean neither starving nor stuffing. Of the two extremes, stuffing is by far the worst. This practice tends to develope the disease, to induce loss of memory, depression of spirits, increased or lessened sleepiness, unusual irritability, epileptiform condition of the nerves, and often transitory coma. In this type of disease the nerve-tissues should be properly fed; and carbonaceous food should by no means be excluded. The brain should not by any means be allowed to remain utterly inactive, but the manner and scope of its exercise should be changed, as cerebral exhaustion and brain-suffering can readily be intensified by utter indolence. New sights and sounds, new friendships and new pursuits—if the pursuits are not too exacting—will often impart new vigor. Sometimes a simple change of posture at night will secure untroubled sleep. If there is undue sleeplessness, the head should lie low on the pillow; if undue sleepiness, it should be kept high.

---

Increased Demand.—The demand for our March number was over three thousand greater than we were able to supply. The chief attraction appeared to be our illustrated article on the new method of constructing boots, shoes, and lasts, invented by Mr. Joel McComber. As many who ordered that number failed to receive it, we have decided to revise and reprint the article which apparently gave to it its chief value. Our issue is a very large one, and we hope to be able to supply all orders for it.

Decline of Life.—Sixty-three is the age when the decline of life commences. Up to that time we are only middle-aged, and ought to be full of health and vigor. But after that epoch let us beware of stumbling-blocks, and watch ourselves narrowly, or we shall never live out our days. A great many things have to do with the duration of life, however, besides our own care of ourselves. Some professions are longer-lived than others—rich people, as a rule, live longer than poor ones, if they live moderately. Clergymen are said to have the longest lives, and medical men the shortest. The general extension of the duration of human life observable for the last three or four generations, may be attributed to sanitary improvements and knowledge; better food, clothing, and ventilation; and in part, also, to the progress of the arts of healing.

---

Dr. Lyon's Tooth-Tablets.—A subscriber in Texas writes that he would like to use the Tablets, since we approve them, but he can not obtain them at the druggist's. We did not suppose that there could be found an apothecary shop in America without this valuable preparation. But we will say to all, that a letter addressed to Dr. I. W. Lyon, Proprietor Tooth Tablets, New York, enclosing fifty cents, will secure a box of this excellent dentifrice by return mail. To those who have not tried the Tablets and would like to test them, a small box will be mailed on receipt of ten cents.

---

Even crumbs are bread, and should not be wasted nor scorned.

